# Treatment Strategies’ Impact on Progression-Free Survival According to RMST Function in Metastatic Colorectal Cancer Patients: A Retrospective Study from Romania

**DOI:** 10.3390/jcm13206174

**Published:** 2024-10-17

**Authors:** Edvina Elena Pirvu, Emilia Severin, Raluca Ileana Patru, Irina Nita, Stefania Andreea Toma, Bianca Elena Croitoru, Adriana Estefa Munoz Groza, Gabriela Marinescu

**Affiliations:** 1 Department of Genetics, “Carol Davila” University of Medicine and Pharmacy, 050474 Bucharest, Romania; 2 Department of Medical Oncology, “Coltea” Clinical Hospital, 030167 Bucharest, Romania; 3 Department of Medical Oncology, Medicover Hospital, 020331 Bucharest, Romania; 4 Department of Medical Oncology, Ponderas Academic Hospital, 014142 Bucharest, Romania

**Keywords:** metastatic colorectal cancer treatment, KRAS mutation, real-world data from Romania

## Abstract

**Background:** This retrospective study investigates the impact of various treatment strategies on progression-free survival (PFS) in patients with metastatic colorectal cancer (mCRC), a significant global health issue. **Methods:** We employed the restricted mean survival time (RMST) to evaluate how different treatments affect PFS over a defined period. The study included 225 patients with mCRC who were treated between 2015 and 2023 at the Oncology Department of Colțea Clinical Hospital in Bucharest. To assign KRAS status, mutation data from exons 2, 3, and 4 of the KRAS gene were required. Eligibility criteria included a confirmed histopathological diagnosis of colorectal adenocarcinoma, a valid RAS mutation test from a solid biopsy, radiological confirmation of stage IV disease by computed tomography, and at least one line of systemic treatment in the metastatic setting. **Results:** Our analysis revealed a small difference in PFS based on KRAS status, but this difference was not statistically significant. Neither sex nor the urban versus rural environment impacted PFS; however, the data indicated that educational level affected survival outcomes. **Conclusions:** Consistent with existing literature, our findings showed no survival benefit from locoregional treatments such as surgery of the primary tumor or curative radiotherapy at diagnosis. In contrast, resection of hepatic metastases was associated with improved survival outcomes.

## 1. Introduction

Colorectal cancer (CRC) is one of the most common malignant diseases worldwide, with significant variations in incidence and prevalence in different regions, according to data provided by the World Health Organization (WHO) [1]. GLOBOCAN confirms that it is the third most commonly diagnosed cancer and the second leading cause of cancer-related deaths, accounting for approximately 10% of all cancer cases and deaths each year, and according to their projections, the global burden of colorectal cancer will increase from 20 million new cases in 2022 to 32.6 million in 2045 [2]. The incidence of CRC is highest in high-income countries such as North America, Western Europe, and Australia. However, many low- and middle-income countries are experiencing rising rates of CRC due to lifestyle changes, aging populations, and improved detection methods [3,4]. Conversely, effective screening programs in high-income countries have contributed to stabilizing or even decreasing CRC incidence rates [5]. Despite these efforts, CRC prevalence remains high, partially due to increasing survival rates and an aging global population, which poses ongoing challenges for healthcare systems worldwide. Statistical data regarding the incidence of the disease and the impact of different treatment options in countries with limited resources are scarce.

RAS mutations are a critical factor in the pathogenesis of colorectal cancer, occurring in approximately 40% of CRC cases. These mutations are serving as a key driver of tumorigenesis through the activation of the RAS/RAF/MEK/ERK signaling pathway [6]. 

Mutations, especially those in codons 12 and 13 of the KRAS gene, are associated with poor prognosis and resistance to anti-EGFR (epidermal growth factor receptor) therapies [7,8]. Consequently, KRAS mutation status is a crucial biomarker for guiding treatment decisions, as the presence of these mutations predicts a lack of response to cetuximab and panitumumab, two commonly used monoclonal antibodies in the management of metastatic CRC [9,10]. As such, testing for KRAS mutations is nowadays standard practice in the management of CRC according to treatment guidelines, helping to personalize therapy and avoid ineffective treatments, thereby improving patient outcomes [11,12]. Research regarding the prevalence and characteristics of KRAS mutations has primarily been conducted in Western countries. The data regarding the characteristics of these mutations and clinical and paraclinical correlations are limited in Romania. 

In our previous work, we tried to better define the population of metastatic colorectal cancer from Romania, by finding correlations between demographic, clinical, and paraclinical variables and outcomes [13]. In this study, we try to define the impact of different treatment strategies on survival. 

Chemotherapy has become a cornerstone in the treatment of colorectal cancer, largely due to research over recent decades showing that its use has significantly improved overall survival (OS) in patients, particularly in those with metastatic disease [14]. Anti-EGFR and anti-VEGF agents are nowadays added to the backbone of chemotherapy, adding survival benefits, but also clinical toxicity and financial challenges [15,16,17].

Although metastatic colorectal cancer is a disseminated disease that needs systemic treatment in order to control it, there is evidence that for selected cases, local treatment (surgery, stereotactic body radiotherapy, radiofrequency ablation, chemoembolization) can improve outcomes [18,19,20].

The aim of this study is to explore the impact of different treatment strategies in a real-world population of patients with metastatic colorectal cancer KRAS wild type and a KRAS mutant. We will use the restricted mean survival time (RMST) to assess these relationships, summarizing treatment effects based on progression-free survival (PFS) over a specific time frame.

## 2. Materials and Methods

We conducted a retrospective, observational, non-randomized study with a cross-sectional design on a cohort of 225 adult patients diagnosed with stage IV colorectal adenocarcinoma. The study, ethically approved by the local Ethics Committee on 9 November 2021, was carried out at the Colțea Clinical Hospital in Bucharest, Romania, between 1 January 2015, and 1 February 2023. All procedures adhered to the ethical standards set forth in the Helsinki Declaration. 

### 2.1. Patient Selection and Inclusion Criteria

To be included in the study, patients needed to meet the following criteria:

- A confirmed histopathological diagnosis of colorectal adenocarcinoma.
- A valid KRAS mutation test performed on a solid biopsy.
- Radiological confirmation of stage IV disease via computed tomography.
- To have received at least one line of systemic treatment for metastatic disease.

### 2.2. KRAS Mutation Testing

KRAS mutation testing involved extracting DNA from the sample using the QIAmp DNA FFPE tissue kit or QIAmp DSP DNA Mini kit and detecting mutations in exons 2, 3, and 4 of the KRAS gene using a targeted resequencing assay (Ion AmpliSeq NGS Panel, Thermo Fisher Scientific, Romont, Fribourg, Switzerland). Sequencing was conducted on the Ion Gene Studio S5 Prime System (Thermo Fisher Scientific), with a detection limit of 2–5% mutant allelic content depending on the genomic region analyzed.

### 2.3. Clinical Staging

Clinical staging was performed according to the TNM classification system, which assesses tumor size, nodal status, and metastasis categories at the time of diagnosis.

### 2.4. Statistical Analysis

Descriptive statistical analysis was conducted on the variables included in the study. For continuous variables, measures of central tendency were calculated using the mean and median, while measures of variability were assessed using standard deviation (SD), minimum, maximum, and the range (difference between maximum and minimum values). The inferential analysis employed methods such as time-to-event survival analysis. Specifically, the Kaplan–Meier estimator was used to graphically represent survival curves and calculate the mean survival and restricted mean survival time (RMST) statistics. To provide a more comprehensive analysis, RMST statistics were compared at multiple time points on the survival curves, using the quartiles of the follow-up period at 17, 29, and 46 months. Hazard ratios (HR) were also calculated. 

RMST represents the average time a patient survives (or remains progression-free) up to a specific time point, known as the restriction time (τ, or “tau”). It is calculated by taking the area under the Kaplan–Meier survival curve (which plots survival probabilities over time) up to the time point τ. This gives the expected survival time within the restricted period. RMST focuses on survival within a specific timeframe, rather than the entire duration of follow-up. This restriction avoids problems caused by late, unstable data points or long follow-up periods where fewer patients are still under observation. The RMST is essentially the cumulative survival time (or progression-free time) for all patients within the defined timeframe. It provides an intuitive measure: “How long, on average, patients are expected to live (or remain progression-free) within τ years?” Unlike the hazard ratio, which gives a relative risk of an event (such as disease progression), RMST is expressed in time units (months or years), making it easier for clinicians and patients to interpret. Mathematically, RMST is the integral of the survival function (S(t)) from 0 to τ:

RMST(τ) = ∫0τS(t)dtRMST(τ) = \int_0^τ S(t) dtRMST(τ) = ∫0τS(t)dt

where S(t) is the probability of surviving (or remaining progression-free) at time t.

It is calculated as the area under the survival curve, from time 0 to τ. If τ is 2 years, RMST will reflect the average survival time (or time without progression) for patients within those 2 years.

The significance level (α) for the analysis was set at 0.05, with values below this threshold considered statistically significant. The statistical analysis was performed using R software, version 4.0.2 (Copyright © 2020 The R Foundation for Statistical Computing, R Core Team, 2020). The R packages used included survival, survminer, survRM2, and gtsummary [21,22,23,24,25]. 

### 2.5. Limitations

This study has several limitations:

- The retrospective design and relatively small patient cohort.
- The 7-year study period, during which patients treated after 2020, had access to newer third-line therapies not available earlier, potentially affecting survival outcomes.
- Immunotherapy and anti-BRAF agents were not reimbursed at the time of study accrual.
- The limited availability of locoregional treatments across different centres in Romania may affect the representativeness of the cohort.
- Mismatch repair deficiency/microsatellite instability tests, as well as NRAS and BRAF testing, were not systematically performed due to reimbursement issues, and their impact was not evaluated.

## 3. Results

### 3.1. Demographic Characteristics and Prevalence of KRAS Mutation

The mean age of the 225 patients included in the study was 64 years (range 31–82 years, standard deviation = 9.85).

There was a slight sex disbalance with 126 male versus 99 female patients. The complete demographic characteristics of the two study groups are presented in Table 1.

**Figure 1:**
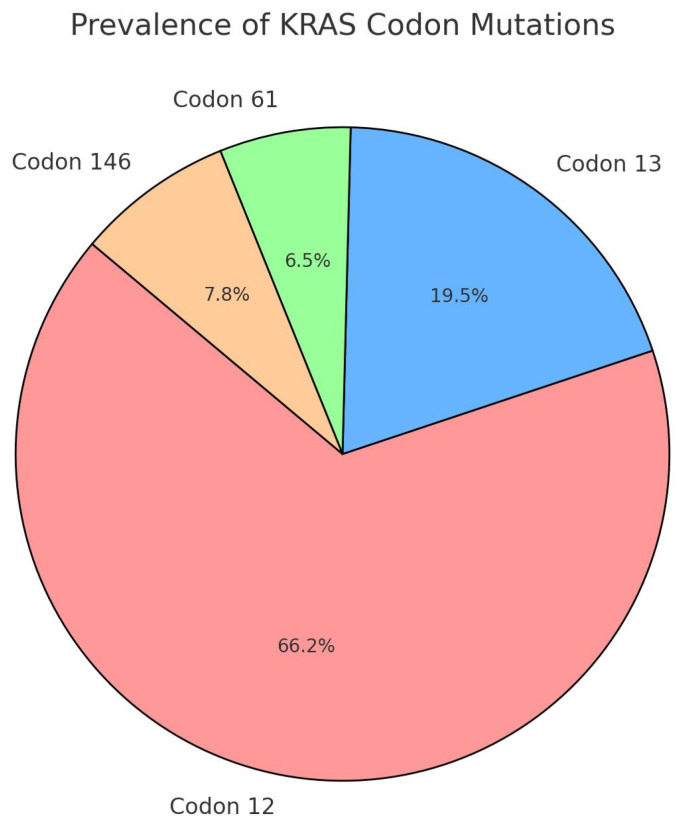
Prevalence of KRAS codon mutations.

In this retrospective study, the overall *RAS* mutation prevalence in mCRC patients was 39.11% (95% confidence interval (CI): [33.07–45.15%]). In total, 11 patients had multiple mutations. For the 77 patients with unique mutations, the following distribution was observed: *KRAS* codon 12 (prevalence 22.67% [17.20–22.14%]); *KRAS* codon 13 (6.67% [3.41–9.93%]); *KRAS* codon 61 (2.22% [0.30–4.15%]; KRAS codon 146 2.67% [0.57–4.77%]. The distribution of mutations is visually represented in Figure 1.

### 3.2. Survival Analysis

**Figure 2:**
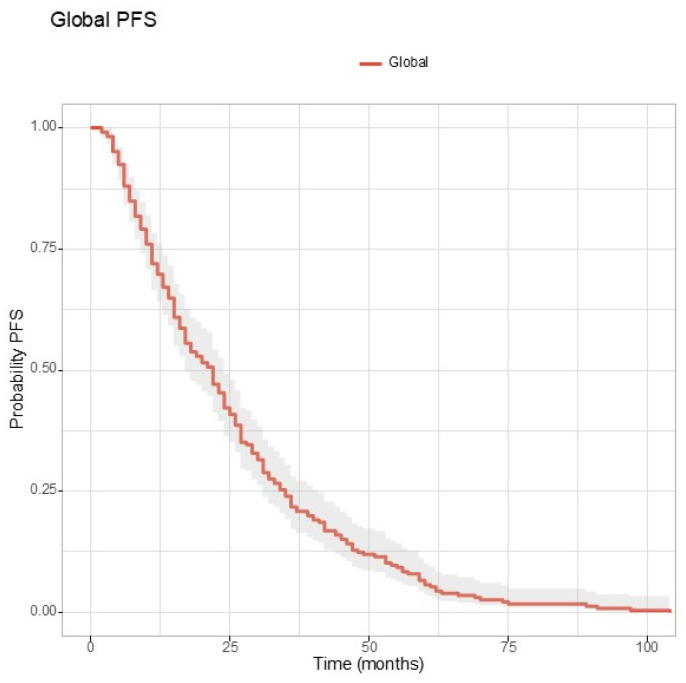
The Kaplan–Meier curve for global progression-free survival.

The global progression-free survival (PFS) for the entire study population was 25.60 months, as visualized in Figure 2.

During the study observation period, all the patients progressed. As presented in Table 2, the small PSF difference between the two groups did not reach statistical significance.

The PFS analysis did not find a statistically significant difference for the female versus male population and rural versus urban patients. The level of education influenced PFS; patients with the lowest education had the fastest disease progression (Table 3).

**Figure 3:**
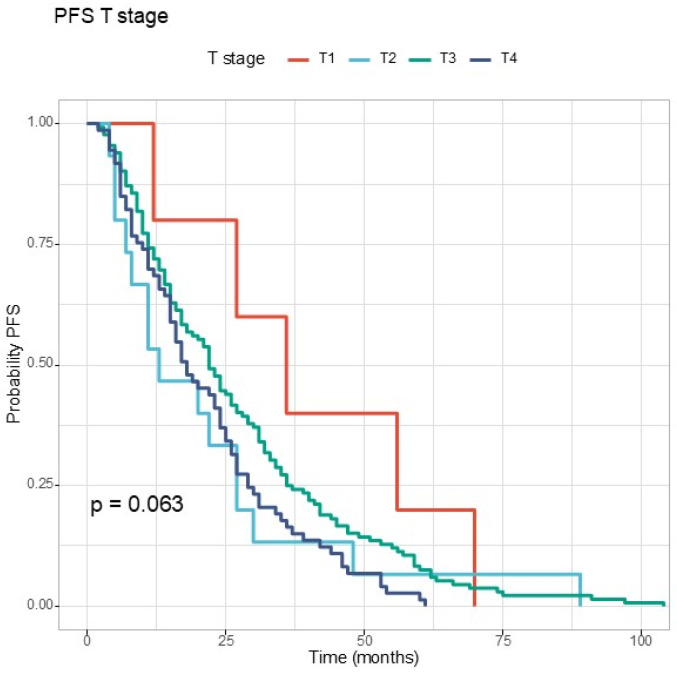
The Kaplan–Meier curve for progression-free survival according to T stage.

**Figure 4:**
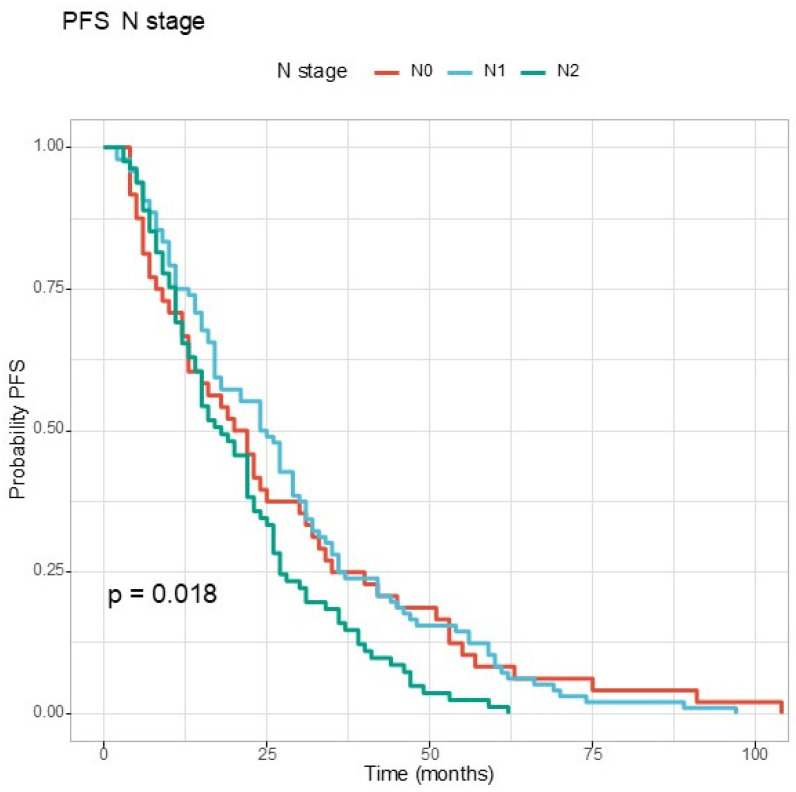
The Kaplan–Meier curve for progression-free survival according to N stage.

Regarding the clinical stage, for T stage, the *p* value was at the limit of statistical significance (Figure 3). For N stage, the difference between the three survival curves can be observed in Figure 4. Patients with N2 disease at diagnosis had the shortest PFS.

The presence of metastasis from diagnosis did not influence PFS (Table 4).

The correlation between treatment strategies and PFS was also analyzed. Surgery of the primary tumor or curative radiotherapy at diagnosis did not influence PFS, as illustrated in Table 5 and Table 6 below.

**Figure 5:**
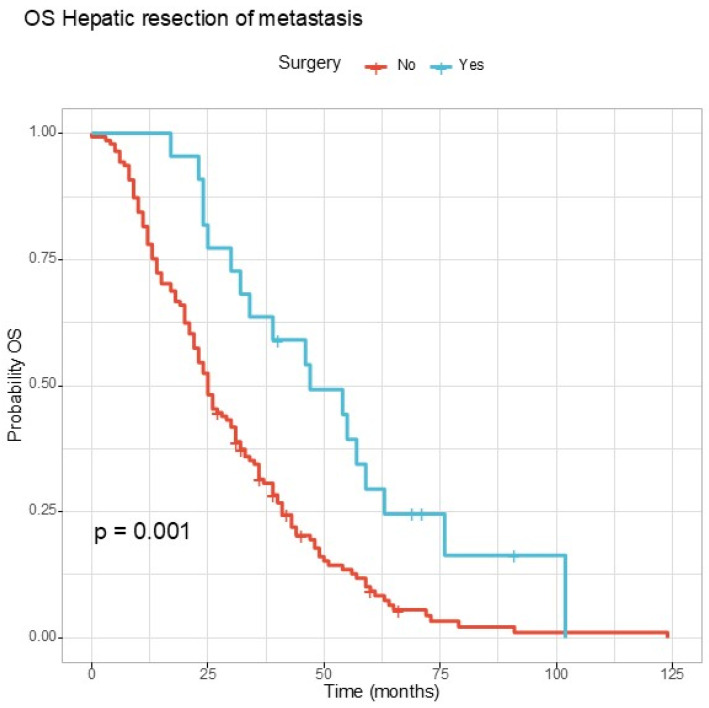
Overall survival according to surgery for hepatic metastasis.

The role of the hepatic resection of metastasis was investigated. Mortality was higher in patients who could not benefit from hepatic resections and median overall survival was approximately two times lower, the differences reaching statistical significance in the log-rank test. The median OS reached 47 months for the group that benefited from the resection of hepatic metastasis versus 25 months for the group that did not have surgery. The results are depicted in Figure 5.

The RMST comparison for the entire period is illustrated in Table 7. For patients that had hepatic surgery for metastasis, RMST was 22 months longer, reaching statistical significance.

**Figure 6:**
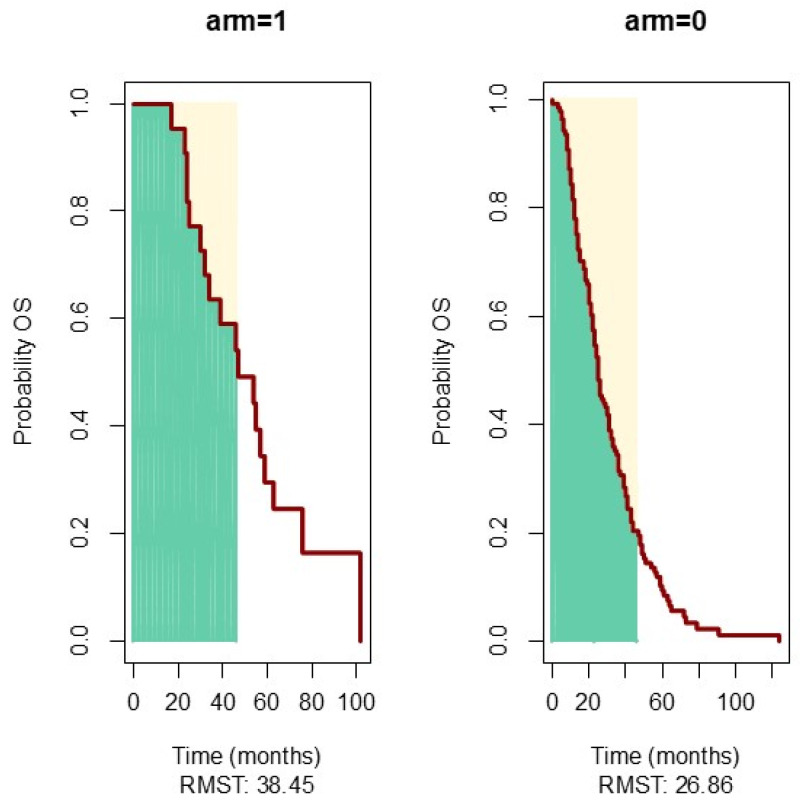
RMST comparison for 46 months of Arm 1 patients with resection of hepatic metastasis. Arm 0: patients without resection of hepatic metastasis. Green shaded area: Restricted Mean Survival. Time Yellow shaded area: The period beyond 46 months where the data is no longer used for RMST calculation. Red line: the Kaplan-Meier survival curve.

For patients that benefited from hepatic resection, RMST for a 46-month interval was 11 months longer, as illustrated in Figure 6.

In order to evaluate the impact of chemotherapy agents, a multiple Cox model was used as represented in Table 8. Patients that received an oxaliplatin-based regimen had a death hazard ratio 1/3 lower, while for irinotecan the death hazard ratio was reduced by half. The utilization of both molecules is associated with effects that are statistically significant and that are independent of the use of other associated molecules.

## 4. Discussion

Colorectal cancer represents a significant global public health challenge, being the third most diagnosed cancer in men and the second most in women according to the GLOBOCAN database [26]. Unfortunately, investigating the burden of metastatic disease from colorectal cancer and having real-world data that can better shape treatment strategies is challenging due to limited cancer registry recordings. In Romania, the National Cancer Registry is not yet completely functional, and even real-world data are limited.

This study aims to address this gap by providing insights into the impact of different treatment strategies on survival outcomes in a Romanian cohort. We chose to use the restricted mean survival time, a statistical measure used in survival analysis to evaluate treatment effects over a specific time period. It is an alternative to the commonly used hazard ratio (HR) and is particularly useful when analyzing time-to-event data, such as progression-free survival (PFS) or overall survival (OS) in clinical trials [27]. 

The advantages of RMST are the following:

○ RMST is useful when the assumption of proportional hazards (required for hazard ratios) is violated. HR assumes that the risk of an event is constant over time, which may not always be true [28].
○ RMST is easier to understand since it gives an average survival time rather than a relative risk.
○ RMST allows the comparison of two or more treatment strategies by comparing the average survival time within the chosen timeframe. For instance, a higher RMST value for one treatment group indicates that patients, on average, live longer without disease progression during the restriction period [29].
○ It gives meaningful results even when there are limited follow-up data. For example, if not all patients have experienced the event (e.g., death or progression), RMST can still provide insights up to a certain point.

In metastatic colorectal cancer, RMST has been used to evaluate progression-free survival and overall survival across different treatment strategies for KRAS wild-type and KRAS mutant patients. Studies show that KRAS mutations negatively impact the response to certain therapies, particularly anti-EGFR treatments like cetuximab, while patients with KRAS wild-type benefit more from targeted therapies like bevacizumab combined with chemotherapy [30]. RMST can provide a clear, time-based comparison between treatment outcomes, highlighting differences in survival under various regimens. 

Regarding the study population, the KRAS mutation prevalence was consistent with the literature, with almost 40% of CRC cases being KRAS mutant and with a distribution of mutations that is similar to the results reported worldwide [31,32]. A small difference in progression-free survival according to KRAS status was observed, but it did not reach statistical significance. 

Our findings regarding the influence of socio-economic factors on survival outcomes are noteworthy. While sex and urban versus rural residence did not significantly impact PFS, education level did. This aligns with global research that socio-economic factors can affect cancer prognosis due to disparities in access to care and early diagnosis [33].

This suggests that targeted interventions aimed at improving access to early detection and treatment for socio-economically disadvantaged populations could enhance survival outcomes.

Regarding treatment strategies, our study confirms existing literature indicating that locoregional treatments, such as surgery of the primary tumor or curative radiotherapy at diagnosis, do not offer significant survival benefits for asymptomatic patients [12,34].

In contrast, the resection of hepatic metastases showed a substantial improvement in RMST, emphasizing the importance of liver-directed therapies in selected cases. This highlights the need for increased accessibility to hepatic surgery and embolization in Romania. Enhanced infrastructure for hepatic units and multidisciplinary evaluations could improve outcomes for patients with liver metastases.

Patients with colorectal cancer and synchronous liver metastases represent a complex group due to the high disease burden and diverse presentations and are nowadays candidates for local treatment at the hepatic level by surgery or different types of embolization [35,36].

Liver resection improves survival in colorectal cancer patients with limited liver metastases primarily because it allows for the complete removal of metastatic disease, which significantly reduces the tumor burden. Surgical resection provides the only potentially curative treatment for these patients. Studies have shown that patients who undergo liver resection have a 5-year survival rate of around 40–60%, compared to less than 5% for patients who are treated with chemotherapy alone. The procedure enables the removal of isolated liver metastases that may not be adequately controlled by systemic therapies, offering the possibility of prolonged disease-free survival or even cure in a subset of patients. Moreover, advances in surgical techniques and perioperative management have reduced operative risks, further enhancing outcomes for patients who undergo this procedure [37,38].

In selected cases, this strategy can improve survival outcomes, as confirmed also in our patients, with an RMST 22 months longer, reaching statistical significance for the patients that had liver resections. Unfortunately, hepatic surgery and embolization have limited accessibility in our country. We hope that these data will motivate policymakers to build a network that will increase the addressability of patients to hepatic units and multidisciplinary evaluation in order to select the candidates that will benefit most. There are three main surgical strategies that proved to be efficient: the traditional staged approach, the combined approach, and the liver-first reverse approach [39]. Recently, the combined and reverse approaches have been used more frequently, with the traditional strategy reserved for cases with tumor obstruction [40]. Most patients should receive preoperative chemotherapy, and treatment must be individualized based on the patient’s condition.

Regarding chemotherapy options, we noticed a positive effect on survival for both oxaliplatin and irinotecan associated with fluoropyrimidine, the backbone for the first-line chemotherapy treatment that has been used for decades and that is confirmed by numerous trials [14,41]. The choice of chemotherapy remains a cornerstone in the management of metastatic CRC, and ongoing research into optimizing these regimens continues to be critical.

This study is not without its limitations. The retrospective design of the study inherently introduces limitations, as it relies on existing medical records, which may lack uniformity in data collection and completeness. This can result in inconsistencies, missing information, or inaccuracies that could influence the study’s outcomes. Additionally, retrospective studies are more susceptible to selection bias because the patients included are not randomized or prospectively selected, which can affect the representativeness of the cohort. A relatively small patient cohort further exacerbates these issues by reducing the statistical power to detect meaningful differences or associations. Smaller sample sizes also limit the ability to generalize findings to a broader population, as the results may be influenced by chance or unique characteristics of the sample, rather than reflecting true population-level effects.

Also, the fact that the study followed patients for a long period of time in order to analyze survival outcomes can also be a limitation because significant advancements in treatment paradigms have occurred. Notably, patients treated after 2020 had access to newer third-line therapies, such as advanced immunotherapies and targeted therapies, that were not available to those treated earlier in the study period. This discrepancy could create a temporal bias, as survival outcomes may be improved in the more recent cohort due to access to these newer, more effective treatments. As a result, the comparison of survival outcomes across the entire study period may not accurately reflect the true impact of earlier treatment regimens, and the overall survival rates might be skewed in favor of patients treated after the introduction of these novel therapies. This uneven access to evolving treatments makes it difficult to draw definitive conclusions about the effectiveness of specific therapeutic approaches across the entire cohort.

Additionally, the lack of systematic mismatch repair deficiency/microsatellite instability testing, as well as NRAS and BRAF testing, may have influenced our findings. These factors were not systematically evaluated due to reimbursement constraints and may represent a gap in our analysis.

Our findings are consistent with those from other studies on KRAS mutations and treatment strategies for metastatic CRC. 

However, the impact of socio-economic factors on survival outcomes and the benefit of hepatic resection in our cohort align with findings from studies conducted in different populations, underscoring the importance of localized data in shaping treatment guidelines.

Future research should focus on larger, prospective studies to confirm these findings and explore the impact of newer therapies and biomarkers. Investigating the role of mismatch repair deficiency/microsatellite instability and other genetic factors in CRC outcomes could provide a more comprehensive understanding of treatment efficacy. Additionally, research into the implementation of effective screening programs and interventions for socio-economically disadvantaged populations is needed.

The findings of this study highlight the need for personalized treatment strategies based on KRAS mutation status and socio-economic factors. Clinicians should consider these factors when developing treatment plans to improve patient outcomes. Enhanced access to specialized care and treatment options, particularly for patients with hepatic metastases, is crucial.

Establishing a functional cancer registry and improving access to comprehensive cancer care are essential for better resource allocation and improving patient outcomes. Policymakers should prioritize the development of infrastructure for hepatic surgery and multidisciplinary evaluations to address the needs of patients with liver metastases and other complex cases.

## 5. Conclusions

The findings of our study align with global research on the subject and help to identify sub-populations that could benefit from the targeted intervention in order to improve outcomes, such as patients with lower education who have a worse prognosis due to late diagnosis and limited access to care, and patients with hepatic metastases who would clearly benefit from hepatic resection and should be addressed in the specialized center in order to improve their survival. Having a functional cancer registry and real-world studies will enable policymakers to gain a complex picture of the particularities of the disease and they will be able to better distribute resources in order to have a bigger impact on outcomes for patients with metastatic colorectal cancer. 

## Figures and Tables

**Table 1:** Demographic characteristics of the KRAS wild type and KRAS mutant populations.

Variable	KRAS Wild Type (N = 137)	KRAS Mutant (N = 88)	*p* Value
**Sex, *n* (%)**			0.84
Female	61 (45)	38 (43)	
Male	76 (55)	50 (57)	
**Age, Mean (SD)**	64.71 (9.58)	64.97 (10.33)	0.85
**Background, *n* (%)**			
Rural	41 (30)	19 (22)	
Urban	96 (70)	69 (78)	
**Education level, *n* (%)**			0.54
I	42 (31)	21 (24)	
II	59 (43)	41 (47)	
III	36 (26)	26 (30)	
**Family history of CRC, *n* (%)**	4 (2.9)	5 (5.7)	0.32
**Alcohol abuse, *n* (%)**	15 (11)	11 (12)	0.72
**Obesity, *n* (%)**	28 (20)	17 (19)	0.84
**Hypertension, *n* (%)**	55 (40)	38 (43)	0.65
**Diabetes, *n* (%)**	25 (18)	7 (8)	0.031
**T stage, *n* (%)**			0.71
T1	2 (1.5)	3 (3.4)	
T2	10 (7.3)	5 (5.7)	
T3	82 (60)	50 (57)	
T4	43 (31)	30 (34)	
**N stage, *n* (%)**			0.31
N0	32 (23)	16 (18)	
N1	53 (39)	43 (49)	
N2	52 (38)	29 (33)	
**M1 stage at diagnosis, *n* (%)**	89 (65)	64 (73)	0.22
**Primary tumor**			
Rectum, *n* (%)	57 (42)	38 (43)	0.82
Cecum, *n* (%)	22 (16)	11 (12)	0.46
Ascending colon, *n* (%)	3 (2.2)	4 (4.5)	0.44
Transverse colon, *n* (%)	5 (3.6)	6 (6.6)	0.35
Rectosigmoid junction, *n* (%)	17 (12)	12 (14)	0.79
Sigmoid colon, *n* (%)	33 (24)	17 (19)	0.40
**Grade of differentiation, *n* (%)**			0.89
G1	18 (13)	11 (12)	
G2	91 (66)	61 (69)	
G3	28 (20)	16 (18)	

**Table 2:** Progression-free survival according to KRAS mutational status.

Strata KRAS Mutation	Progression N (%)	Mean PFS	Median PFS (95% CI)
**KRAS wild type**	137/137 (100)	24.50	20.00 (16.00 to 25.00)
**KRAS mutant**	88/88 (100)	27.20	22.00 (17.00 to 27.00)

CI, confidence interval.

**Table 3:** Progression-free survival according to the level of education Cox regression.

Predictor	Progression N (%)	Mean PFS	Median PFS (95%)
**Level of education**			
**I**	63/63 (100)	20.10	15.00 (11.00 to 22.00)
**II**	100/100 (100)	26.90	23.00 (18.00 to 27.00)
**III**	62/62 (100)	28.90	24.00 (19.00 to 30.00)

CI, confidence interval.

**Table 4:** Progression-free survival according to metastatic status at diagnosis.

Strata Metastasis	Progression N (%)	Mean PFS	Median PFS (95% CI)
**No**	72/72 (100)	22.70	16.00 (13.00 to 23.00)
**Yes**	153/153 (100)	26.90	24.00 (20.00 to 26.00)

CI, confidence interval.

**Table 5:** Progression-free survival according to surgery of the primary tumor.

Strata Surgery of the Primary Tumor	Progression N (%)	Mean PFS	Median PFS (95% CI)
**No**	47/47 (100)	23.10	18.00 (13.00 to 26.00)
**Yes**	178/178 (100)	26.20	22.00 (17.00 to 26.00)

CI, confidence interval.

**Table 6:** Progression-free survival according to curative radiotherapy of the primary tumor.

Strata Curative Radiotherapy of the Primary Tumor	Progression N (%)	Mean PFS	Median PFS (95% CI)
**No**	193/193 (100)	25.60	22.00 (17.00 to 25.00)
**Yes**	32/32 (100)	25.20	22.50 (15.00 to 32.00)

**Table 7:** RMST comparison according to hepatic surgery for metastasis.

RMST Resection of Metastasis	RMST No Resection of Metastasis	*p* Value	Difference (95% CI)
**53.01**	30.60	25.60	22.00 (17.00 to 25.00)

**Table 8:** Multiple Cox model regarding the impact of different treatment molecules.

Predictor	N	N Deaths	HR (95% CI)	*p* Value
Oxaliplatin				
No	71	66	-	
Yes	153	139	0.36 (0.17 to 0.74)	0.006
Irinotecan				
No	157	143	-	
Yes	67	62	0.43 (0.20 to 0.90)	0.025
Monoclonal antibodies				
No	71	68	-	
Yes	153	137	1.04 (0.25 to 4.33)	0.96
Anti-VEGF				
No	138	128	-	
Yes	86	77	0.76 (0.18 to 3.25)	0.71
Anti-EGFR				
No	157	145	-	
Yes	67	60	0.85 (0.20 to 3.59)	0.83

HR = Hazard ratio, CI = confidence interval, anti-VEGF = monoclonal anti-vascular epithelial growth factor antibodies, anti EGFR = monoclonal anti-epidermal growth factor receptor antibodies.

## Data Availability

Data are available on request due to ethical restrictions. The data presented in this study are available on request from the corresponding author. The data are not publicly available due to the policy of Colțea Clinical Hospital to have the approval of the Ethics Committee for each new research study.
